# The Role of microRNAs in the Infection by *T. gondii* in Humans

**DOI:** 10.3389/fcimb.2021.670548

**Published:** 2021-05-14

**Authors:** Geraldo Magela de Faria Junior, Fernando Henrique Antunes Murata, Hernan Alejandro Lorenzi, Bruno Bello Pede Castro, Letícia Carolina Paraboli Assoni, Christiane Maria Ayo, Cinara Cássia Brandão, Luiz Carlos de Mattos

**Affiliations:** ^1^ Immunogenetics Laboratory, Molecular Biology Department, Faculdade de Medicina de São José do Rio Preto (FAMERP), São José do Rio Preto, Brazil; ^2^ Beltsville Agricultural Research Center, Animal Parasitic Diseases Laboratory, United States Department of Agriculture, Agricultural Research Service, Beltsville, MD, United States; ^3^ Department of Infectious Diseases, J. Craig Venter Institute, Rockville, MD, United States; ^4^ Department of Preventive Veterinary Medicine and Animal Health, Faculty of Veterinary Medicine, University of São Paulo, São Paulo, Brazil

**Keywords:** microRNAs, immune system, *toxoplasma gondii*, communicable diseases, toxoplasmosis

## Abstract

MicroRNAs are molecules belonging to an evolutionarily conserved family of small non-coding RNAs, which act on post-transcriptional gene regulation, causing messenger RNA (mRNA) degradation or inhibiting mRNA translation into proteins. These molecules represent potential biomarkers for diagnosis, non-invasive prognosis, and monitoring the development of the disease. Moreover, they may provide additional information on the pathophysiology of parasitic infections and guide strategies for treatment. The Apicomplexan parasite *Toxoplasma gondii* modifies the levels of microRNAs and mRNAs in infected host cells by modulating the innate and adaptive immune responses, facilitating its survival within the host. Some studies have shown that microRNAs are promising molecular markers for developing diagnostic tools for human toxoplasmosis. MicroRNAs can be detected in human specimens collected using non-invasive procedures. changes in the circulating host microRNAs have been associated with *T. gondii* infection in mice and ocular toxoplasmosis in humans. Besides, microRNAs can be amplified from samples using sensitive and molecular-specific approaches such as real-time PCR. This review presents recent findings of the role that microRNAs play during *T. gondii* infection and discuss their potential use of these small nuclei acid molecules to different approaches such as laboratory diagnosis, modulation of cell and tissue infected as other potential applications in human toxoplasmosis.

## Introduction

MicroRNAs are small non-coding RNAs acting on post-transcriptional regulation of gene expression, causing messenger RNA (mRNA) degradation or blocking mRNA translation (Glinge et al., 2017; Malla et al., 2019). Since microRNAs are present in serum and plasma (Chen et al., 2008; Chim et al., 2008; Gilad et al., 2008; Lawrie et al., 2008; Mitchell et al., 2008; McDonald et al., 2011), urine (Hanke et al., 2010; Gidlöf et al., 2011; Glinge et al., 2017), and other body fluids (Park et al., 2009; Weber et al., 2010; Glinge et al., 2017), the interest to explore them as potential biomarkers for diagnosis, non-invasive prognosis, and monitoring the development of the disease (Li et al., 2019). Besides, they may provide additional information on the pathophysiology of disease and guide treatment strategies (Lu et al., 2005; Perron et al., 2007; Gilad et al., 2008; Wang et al., 2009; D’Alessandra et al., 2010; Tijsen et al., 2010; Wang et al., 2010; Glinge et al., 2017; Hu et al., 2018).

The expression of microRNAs has also been reported in infection by Apicomplexan microorganisms, mostly obligatory intracellular parasites infecting animals and humans and causing parasitic diseases of significant public health impact (Cavalier-Smith, 1993). Some of these parasitic diseases are caused by *Plasmodium falciparum*, *Plasmodium vivax*, *Cryptosporidium parvum*, and *Toxoplasma gondii* (Lüder and Gross, 2005; World Health Organization, 2015) which are commonly reported in outbreaks in Brazil (Judice et al., 2016). These parasites can hijack host gene expression, modulating immune response pathways, including those involved in apoptosis and cytokine production (Judice et al., 2016). Thus, the intracellular modulation of host gene expression improves the ability of Apicomplexans to infect and proliferate in target cells such as epithelial (Deng et al., 2004; Lüder and Gross, 2005; McDonald et al., 2013), liver (Prudêncio et al., 2006; Sturm et al., 2006), erythrocytes (Gazzinelli et al., 2014), and some immune cells (Leng et al., 2009).

Recently, it was demonstrated that the microRNAs miR-155-5p and miR-29c-3p are up-expressed, and the miR-21-5p and miR-125b-5p are down-expressed in acute ocular toxoplasmosis, in comparison to asymptomatic individuals (Pereira et al., 2019). These data open the opportunity to explore the up-and down-expression of microRNA as potential tools to investigate many aspects of this parasitic disease. The review discusses the importance of microRNAs in the infection by *T. gondii* and toxoplasmosis, a disease of significant concern to public health worldwide.

## microRNA – Definition, Biogenesis, and Function

MicroRNAs are 18-22 nucleotides long non-coding RNAs that act on post-transcriptional gene regulation causing degradation of messenger RNA (mRNA) or inhibiting its translation into proteins (Glinge et al., 2017; Malla et al., 2019). MicroRNA influence many cellular processes, including cell proliferation, differentiation, migration, apoptosis, angiogenesis, and carcinogenesis (Malla et al., 2019).

The first microRNA described, named lin-4, was identified by Lee et al., 1993 in the nematode *Caenorhabditis elegans* (Lee et al., 1993). It was later described in eukaryotes, including humans (Tang et al., 2018). Subsequent studies identified 28,000 mature microRNAs by sequencing (Malla et al., 2019), and 2,500 were found in humans as sotirage in mirbase (http://www.mirbase.org/) (Glinge et al., 2017) of which 60% regulate protein-encoding genes (Friedman et al., 2009; Glinge et al., 2017). Details can be found in a recent review by Judice et al. (2016).

The study and processing of microRNAs as biomarkers requires attention since it’s fragile structure and stability may be compromised depending on the methods and the biological material used (e.g., blood, serum, plasma, urine) (Wang et al., 2012; Rice et al., 2015; Glinge et al., 2017). Nevertheless, there are reports that microRNAs are stable in serum and plasma samples, and they are resistant to RNAse action, extreme pH, freezing and thawing conditions (Chen et al., 2008; Gilad et al., 2008; Mitchell et al., 2008; Glinge et al., 2017).

**Figure 1:**
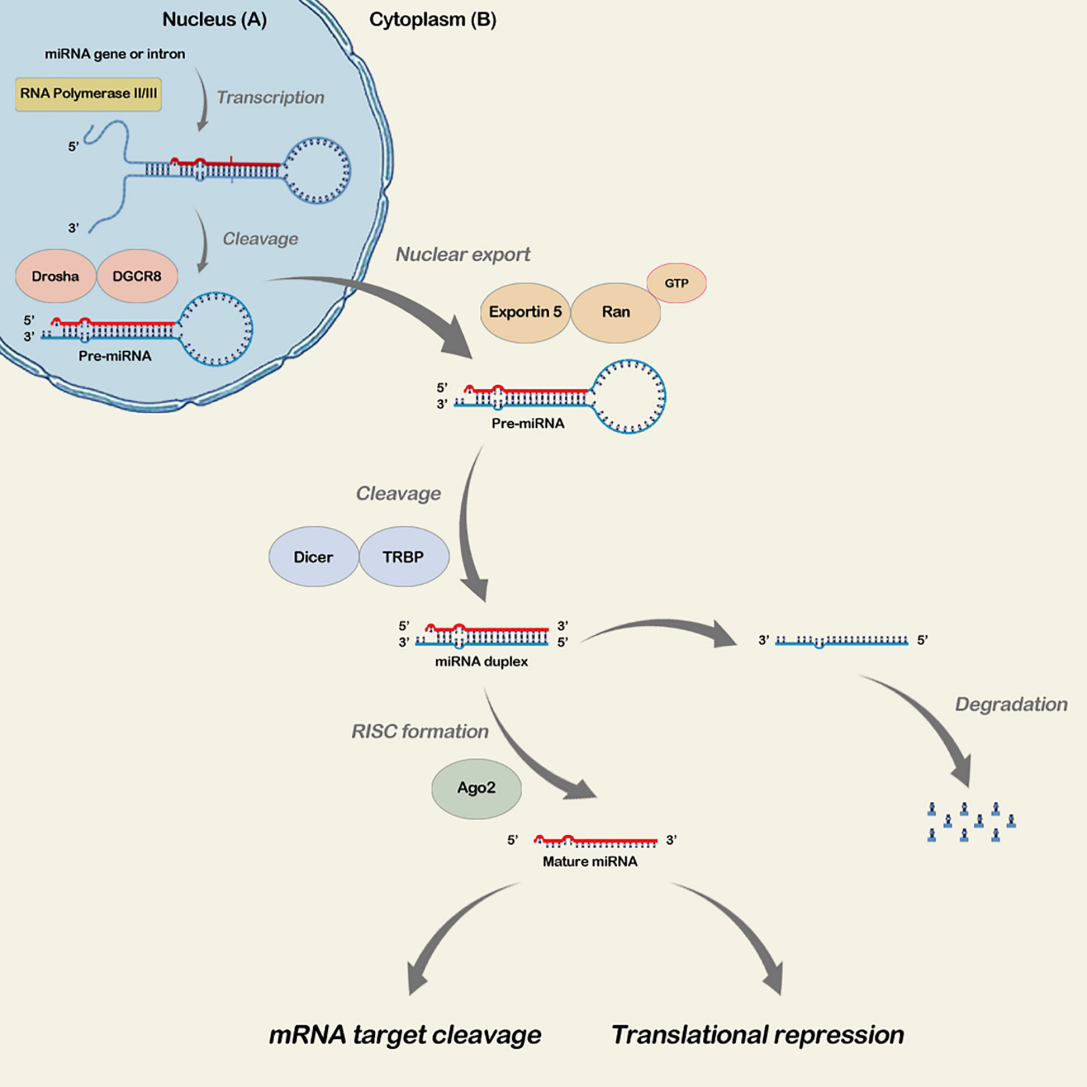
microRNA Biogenesis Steps. In the nucleus, miRNA gene transcription occurs by RNA polymerase III, resulting in the primary double-stranded miRNA (pri73 miRNA). The miRNA precursor (pre-miRNA) formation occurs, which is transported to the cytoplasm through exp5 and the Ran GTP cofactor **(A)**. In the cytoplasm, pre miRNA is processed by Dicer RNAse, resulting in a double-stranded RNA, mature RNA and microRNA-5p. The second is degraded, and the mature microRNA binds to Argonaut protein (AGO) and forms the RNA-induced silencer complex (RISC). Mature microRNAs incorporate into RISC, regulating gene expression by mRNA degradation or transductional repression **(B)**.

MicroRNA expression is complex and begins in the cell nucleus. The miRNA gene transcription by the action of RNA polymerase II results in the formation of a double-stranded primary miRNA (pri-miRNA) with a tail at its 5’ end and a poly-A tail at the 3’ end (Filella and Foj, 2017; Malla et al., 2019). Pri-miRNAs then give rise to a hairpin structure that mates with a microprocessor (500-650 kDa). This structure has an endonuclease RNAse III (Drosha) and an essential cofactor (DGCR8/Pasha), which combine and form the precursor miRNA (pre-miRNA) that is then transported to the cytoplasm by a nuclear export protein called Exportin-5 (exp5) and the Ran GTP cofactor (Malla et al., 2019). In the cytoplasm, the pre-miRNA is processed by the Dicer RNase, resulting in a double-stranded RNA of approximately 22 nucleotides. One strand becomes the mature microRNA, while the other, a microRNA-5p, is degraded (Filella and Foj, 2017; Malla et al., 2019). Subsequently, the mature microRNA binds to the Argonaut protein (AGO) and forms the RNA-induced silencer complex (RISC) (Chan et al., 2005; Murakami et al., 2006; Filella and Foj, 2017; Malla et al., 2019). Mature microRNAs are incorporated into RISC to regulate gene expression by mRNA degradation or translational repression (Murakami et al., 2006) (**Figure 1**).

## *Toxoplasma gondii* Infection and Host Immune Response

*Toxoplasma gondii (T. gondii)*, the etiological agent for toxoplasmosis, is an intracellular parasite that infects nucleated cells from all warm-blooded animals (Li et al., 2019; Hou et al., 2019). It has three infectious stages known as tachyzoite, bradyzoite, and sporozoites (within oocysts) (Dubey et al., 1998). Tachyzoites are crescent-shaped, with a pointed end in the anterior region and rounded in the posterior region. This mobile stage can invade nucleated cells and multiply by repeated endodyogeny within parasitophorous vacuoles within any cell of the intermediate host and in non-intestinal epithelial cells of the definitive host (Robert-Gangneux and Dardé, 2012). Bradyzoites, also called cystozoites, is the form found in tissue cysts of intermediate hosts and multiply slowly within a tissue cyst (Dubey et al., 1998). Infected felines can eliminate unsporulated oocysts with faeces to the environment. Under ideal temperature and humidity conditions, oocysts sporulate, forming two sporocysts containing four sporozoites each. The oocyst wall is characterised by a multilayered structure protecting the parasite against physical and chemical damage, allowing the parasite to survive for long periods in the environment (Dubey et al., 1998; Robert-Gangneux and Dardé, 2012).

*T. gondii* is horizontally transmitted by ingestion of contaminated water and food and vertically from mother to child during pregnancy (Robert-Gangneux and Dardé, 2012; Murata et al., 2016). In most cases, the infection by *T. gondii* is asymptomatic (Lima and Lodoen, 2019). However, the disease is commonly severe in immunocompromised individuals and neonates (Robert-Gangneux and Dardé, 2012). A small portion of immunocompetent individuals may also develop symptoms (Robert-Gangneux and Dardé, 2012). Immunosuppressed individuals commonly develop neurological symptoms and encephalitis. However, if the infection is acquired during pregnancy, it can result in severe or fatal toxoplasmosis (Robert-Gangneux and Dardé, 2012). The severity of the disease is associated with conditions such as the period of gestation, type of strain, dose and host immune system (Dubey et al., 2012; Murata et al., 2016), and congenital toxoplasmosis may lead to visual and hearing complications and cognitive impairments (Li et al., 2019).

The human immune response against *T. gondii* infection includes the production of IL-12 by neutrophils, dendritic cells and macrophages (Bliss et al., 1999; Aldebert et al., 2007). These cytokines are crucial for host resistance, blocking parasite replication and increasing the degradation of tryptophan (Pfefferkorn, 1984; Lima and Lodoen, 2019).

*T. gondii* uses sophisticated strategies to infect the host cell. The parasite releases proteins from organelles called rhoptries and dense granules, signalising host cells and their transcriptional responses (Lima and Lodoen, 2019; Tuladhar et al., 2019). By this mechanism, the parasite can manipulate host signalling pathways, modulating the release of cytokines and consequently compromising an effective host immune response against the parasite.

Three types of *T. gondii* strains called type I, type II and type III carrying different virulence factors were identified in mouse models. Studies have shown that the type I strain is the most virulent while type II and III strains are avirulent (Howe et al., 1996; Mordue et al., 2001). Type I strain can cause a lethal infection in mice at a dose of 1 parasite, while type II and III strains have a lethal media dose equal to or higher than 10^5^ (Sibley and Boothroyd, 1992). An experimental study identified several characteristics that may correlate with virulence in a host, including phenotyping difference in growth, migration, and transmigration, with type I strain growing faster and with migration abilities greater than type II and III strains (Barragan and Sibley, 2003). The type I strain virulence correlates with the immune response inducing a more potent TH1-inflammatory response than Type II or III. Genetic studies have identified secretory proteins discharged from apical organelles, called rhoptries (ROPs), as the determinant of acute virulence in type 1 strain (Taylor et al., 2006; Saeij et al., 2007).

A study conducted by Saeji et al., 2006 showed that human and mouse cells response to parasite infection depends on *T. gondii* strain (e.g., types I, II and III). Types I and III (encoding ROP16 allele *ROP16*_I/III_) cause direct and prolonged phosphorylation of host transcription factors STAT3 and STAT6 (Saeij et al., 2006). As a result, there is a decrease in the production of IL-12 in macrophages (Saeij et al., 2006; Saeij et al., 2007; Butcher et al., 2011; Cai and Shen, 2017; Lima and Lodoen, 2019; Tuladhar et al., 2019). Type II strains (carrying a *GRA15*_II_ allele) activate the host transcription factor NF-kB, which leads to the production of pro-inflammatory cytokines in the host cell (Butcher et al., 2005; Lima and Lodoen, 2019; Tuladhar et al., 2019). These effects are caused by parasite rhoptry and dense granule proteins (Saeij et al., 2007; Ong et al., 2010; Yamamoto et al., 2012).

In humans and animals, the immune response mediated by IFN-γ is essential to control acute and chronic infections caused by the parasite (Suzuki et al., 1988; Suzuki et al., 1989). This cytokine can induce a vast transcriptional program (Platanias, 2005), and *T. gondii* infection blocks the positive regulation of many of those IFN-γ-controlled genes (Kim et al., 2007).

Studies in humans have shown that the three types of parasite strains described above can inhibit the transcriptional activity of the STAT1 protein through ROP16 or GRA15 proteins, which activate the NF-kB signalling pathway (Rosowski et al., 2011; Rosowski and Saeij, 2012; Lima and Lodoen, 2019). With the release of IFN-γ, signalling of the JAK/STAT pathway begins, allowing the displacement of the STAT1 homodimers to the cell nucleus, where it interacts with the gamma-activated sequence (GAS) in the DNA to initiate transcription (Sadzak et al., 2008). In response, *T. gondii* inhibits the expression of human genes that respond to IFN-γ, blocking the expression of the JAK/STAT pathway and consequently preventing the separation of STAT1 from the host nuclear DNA (Rosowski et al., 2014).

Studies conducted by Gray et al., 2016 and Olias et al., 2016 revealed two mechanisms used by the parasite to manipulate the host immune system: (i) the inhibitor of STAT1-dependent transcription (TgIST), which is a protein, binds to activated STAT1 dimers in the nucleus of IFN-γ-dependent cells, and (ii) the Mi2/NuRD complex, which can modify the chromatin of cells, blocking IFN-γ-dependent transcriptional mechanisms (Gray et al., 2015; Olias et al., 2016).

**Figure 2:**
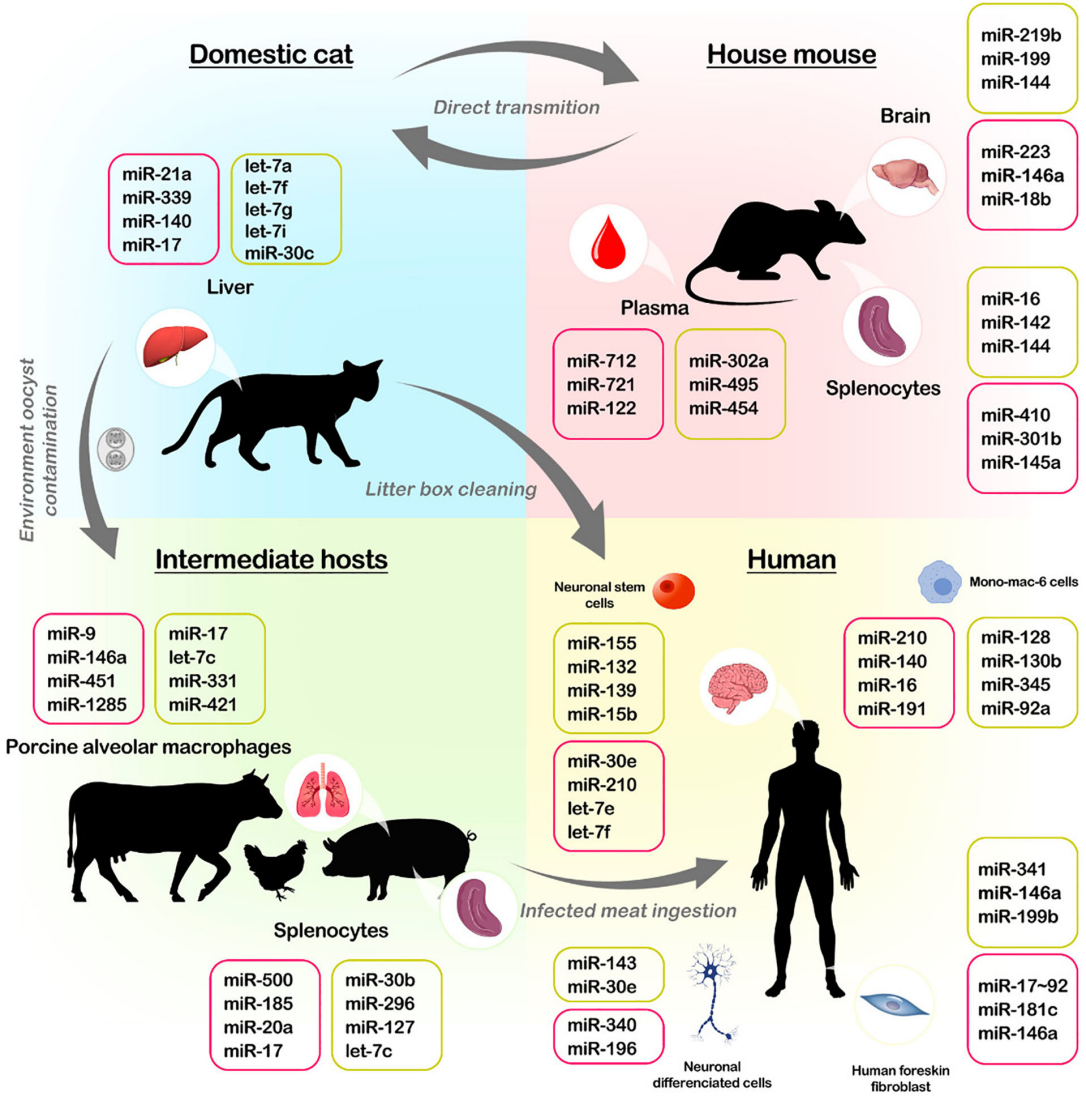
Life cycle of Toxoplasma and host miRNA interaction. *T gondii* has a complex life cycle, in which the parasite can infect felids (domestic cats), farm animals, mice, and even humans. Felids are definitive hosts. Oocysts are released from infected cat faeces and become infected in the environment after sporulate. Infected felids present modifications in microRNA expressions. Humans become infected by ingestion of undercooked meat of infected animals or by the ingestion of soil, water or food contained sporulated oocysts derived from the environment. In the human host, the disease can affect various organs tissues such as skeletal muscle, brain (neuronally differentiated cells and neuronal stem cells) and myocardium, presenting modifications in microRNA expressions. Also, porcine alveolar macrophages and splenocytes samples from infected pigs have modified microRNA profiles. Mice are used mainly to study toxoplasmosis *in vivo* because they may naturally be infected by the parasite and affect multiple organs while changing their microRNA profile. These animals can also be having multiple organs affected by the infection, and it is known that the spleen, plasma, and brain have their miRNA profile altered during the infection. The colour box represents the increase (pink) and decreases (yellow) of miRNA expressions.

The NF-kB signalling pathway is another important pathway deregulated by *T. gondii*, which produces pro-inflammatory cytokines in host immunity (Lima and Lodoen, 2019). It was observed that in Human Foreskin Fibroblast (HFFs) infected with a type I strain of *T. gondii*, phosphorylation of the transcription factor p65/RelA was reduced, preventing translocation to the host cell nucleus and limiting the activation of the NF-kB pathway (Butcher et al., 2011). Besides, the same type I strain inhibited the production of IL-1*β* by human neutrophils, impairing the activity of the NF-kB pathway (Lima and Lodoen, 2019) (**Figure 2**).

## Host microRNAs During *Toxoplasma gondii* Infection

The microRNA blockade of mRNA is fundamental for protection against pathogenic virus and bacteria in plants, insects and animals (Hou et al., 2019). microRNAs interact mainly with the 3’ untranslated region of their target mRNAs, controlling the translation or affecting the transcript stability favouring mRNA degradation (Hou et al., 2019). Some studies have shown that Apicomplexan parasites affect the host microRNA expression profile (Deng et al., 2004; McDonald et al., 2013; Hou et al., 2019). After cell invasion, these microorganisms regulate gene expression of host cells, including those cells of the immune system, such as macrophages and dendritic cells (Leng et al., 2009; Hou et al., 2019). This process contributes to parasite persistence and microbial growth (Blader et al., 2001; Hou et al., 2019).

*T. gondii* infection perturbs the expression of specific host microRNAs, which contributes to efficient parasite replication (Cong et al., 2017) by altering the signalling pathways involved in the defensive response of infected cells (Hakimi and Ménard, 2010). The infection by cysts and tachyzoites lead to altered microRNA expression in mouse brain (Xu et al., 2013) and spleen (He et al., 2016). However, it is unknown whether sporulated oocyst infections also modify the expression levels of microRNAs in animal brains during acute and chronic infection (Hu et al., 2018).

There is evidence that *T. gondii* modulates the expression of important microRNAs (Zeiner et al., 2010). With the activation state of NF-κB, 14% of the host microRNAs are altered in primary fibroblasts 24 hours post-infection (Shapira et al., 2002; Judice et al., 2016). In agreement with these observations, two studies showed that during infection, there is an increased expression of miR-17-92 and miR-106b-25, which regulate the progression of the mammalian cell cycle from G1 to the S phase influencing the apoptosis pathways (Xiao and Rajewsky, 2009; Cai and Shen, 2017) (**Table 1**).

**Table 1:** The role of microRNAs in toxoplasmosis diseases.

microRNA	Disease/Parasite	Effects	References
microRNA-17-92; microRNA-106b-25	Toxoplasma/*T. gondii*	Apoptosis and G1/S cell cycle transition pathways.	(Xiao and Rajewsky, 2009; Cai and Shen, 2017)
microRNA-146a	Toxoplasma/*T. gondii*	NF-κB signalling pathway	(Taganov et al., 2006)
microRNA-30c-1; microRNA-125b-2; microRNA-23b-27b-24-1; microRNA-17-92	Toxoplasma/*T. gondii*	Associated with the anti-apoptosis responses of the host cells.	(Cai et al., 2013)
microRNA-146a	Toxoplasma/*T. gondii*	Cellular response in the host to infection by *T. gondii* and inflammatory response regulator.	(Taganov et al., 2006; Cannella et al., 2014; Saba et al., 2014; Judice et al., 2016)
microRNA-155	Toxoplasma/*T. gondii*	Cellular response in the host to infection by *T. gondii* and required for Cytokine expression by TH17 cells and Treg-Cell homeostasis.	(Cannella et al., 2014; Cai and Shen, 2017)
microRNA-132b	Toxoplasma/*T. gondii*	Influence on *Toxoplasma* encephalopathy.	(Cai and Shen, 2017)
microRNA-712-3p; microRNA-511-5p; microRNA-217-5p	Toxoplasma/*T. gondii*	Possible biomarkers of *T. gondii* infection	(Jia et al., 2014)

The transcription factor NF-κB plays a fundamental role in *T. gondii* immunity, and it is believed that the parasite might use it to modulate innate and adaptive immune responses of the host (Denkers et al., 2004; Mason et al., 2004). *T. gondii* can suppress NF-κB activation (Shapira et al., 2002) by inducing the expression of miR-146a in the host (Taganov et al., 2006). The activation of NF-κB signalling and *STAT3* gene up-regulates the expression of miRNAs miR-30c-1, miR-125b-2, miR-23b-27b-24-1 and miR-17-92 in response to *T. gondii* infection (Cai et al., 2013). (**Table 1**). The expression of the immunomodulatory microRNAs miR-146a and miR-155 was induced in the brains of mice infected with specific *T. gondii* strains (Cannella et al., 2014). Animals infected with a type II strain showed significant induction of miR-146a, an essential regulator of the inflammatory immune response (Taganov et al., 2006; Saba et al., 2014; Judice et al., 2016). It was also observed that the absence of miR-146a expression affects parasitic load, leading to significant differences in IFN-γ production and long-term survival of infected mice (Judice et al., 2016).

The microRNA miR-155 is highly expressed in human and animal Th17 cells (Hu et al., 2013; Escobar et al., 2014) (**Table 1**). Besides, this microRNA is critical for TH17 cell cytokine expression and Treg cell homeostasis. Studies in animals showed that miR-155 is associated with recruiting Treg and CD8+ cells in *T. gondii* infection (Cai and Shen, 2017). microRNAs can also modify innate immune response signalling through pathogen-aware receptors (Fabbri et al., 2013). An example is miR-132b, found in abundance in neural tissue cells and regulated by cyclic AMP-response element-binding (CREB). This microRNA is involved in neurological disorders such as schizophrenia, Alzheimer disease, Parkinson disease and is also involved in *T. gondii* encephalopathy (Miller et al., 2012; Cai and Shen, 2017) (**Table 1**).

Another study in mice by Cai et al. (2014) found three microRNAs (miR-712-3p, miR-511-5p, and miR-217-5p) at the beginning of the infection. According to these authors, the increase in these microRNAs is *T. gondii* specific, and these molecules are predominantly expressed in cells infected with RH and ME49 strains (Miller et al., 2012; Cai et al., 2014). Thus, the expression profile of these microRNAs suggests that they can be used as biomarkers of *T. gondii* infection. Furthermore, other pathogens such as *Plasmodium berghei*, *Plasmodium yoelii*, *Plasmodium chabaudi*, and *C. parvum* do not induce the expression of these microRNAs reinforces the use of microRNAs as potential *T. gondii b*iomarkers (Judice et al., 2016). Therefore, the potential specific expression of miR-712-3p, miR-511-5p and miR-217-5p during *T. gondii* infection makes them excellent candidate biomarkers for diagnosis (Judice et al., 2016).

Recently, two studies carried out in Brazil evaluated the expression of microRNAs in ocular toxoplasmosis and cerebral toxoplasmosis in HIV patients (Pereira et al., 2019). In one of them, the authors reported that the microRNAs miR-155-5p and miR-29c-3p were up-expressed in ocular toxoplasmosis compared to asymptomatic individuals. They also observed that the miR-21-5p and miR-125b-5p were down-expressed in acute ocular toxoplasmosis compared to asymptomatic individuals (Pereira et al., 2019). The other one demonstrated that the miR-21-5p and miR-146a5p were up-expressed in HIV patients with cerebral toxoplasmosis compared with asymptomatic individuals and seronegative individuals. These authors observed that the plasma of HIV patients with cerebral toxoplasmosis CT/HIV and asymptomatic individuals expressed similar levels of miR-29c-3p, miR-155-5p and miR-125b-5p (Pereira et al., 2019). The data showed in these studies demonstrate some specific patterns of miRNAs as potential biomarkers for ocular toxoplasmosis and HIV cerebral toxoplasmosis patients. These findings may help understand the complex parasite-host interaction and diagnosis, prognosis, and therapeutic control in human toxoplasmosis.

## microRNAs for Diagnosis of *T. gondii* Infection in Humans

*T. gondii* infection is usually asymptomatic in healthy individuals. When symptomatic, most infected people develop non-specific symptoms such as fever or cervical lymphadenopathy that can be easily misdiagnosed as the common flu, mononucleosis, etc. (Robert-Gangneux and Dardé, 2012). Ocular toxoplasmosis is the most common complication caused by *T. gondii* infection. However, the infection can be severe or even fatal in immunocompromised individuals (e.g. HIV-infected, solid organs transplant recipients) and by transplacental transmission, the foetus may develop severe stages of the pulmonary disease disseminated and cerebral toxoplasmosis (Robert-Gangneux et al., 2018).

The diagnosis of toxoplasmosis is still based on serology and clinical evaluation (Villard et al., 2016). Serological tests are essential, and most can determine the stage of infection (Dhakal et al., 2015; Murata et al., 2016). It is crucial to monitor the serological status of pregnant women since the parasite can cross the placental barrier and infect the foetus leading in many cases to severe illness and abortion (Robert-Gangneux and Dardé, 2012). Anti-*T. gondii* IgM and IgG antibodies are common biomarkers for the diagnosis of toxoplasmosis. IgM is associated with recent infection and IgG with chronic infection. Although the evaluation of these antibodies differentiates acute from chronically infected patients, antibodies may be persistent or absent in some cases. Therefore, additional tests are necessary to confirm serological results, making the diagnose of *T. gondii* difficult and time-consuming (Goebel et al., 1999; Carmen et al., 2006; Cai and Shen, 2017). IgM antibodies usually appear one week after infection and may drop to undetectable levels within six months. However, in some cases, IgM can be detected for more than one year after infection (Goebel et al., 1999). The evaluation of other acute antibodies like IgA in conjunction with IgM can help diagnose acute toxoplasmosis, especially in neonates (Montoya, 2002; Murata et al., 2016; Pomares and Montoya, 2016). IgG antibodies usually appear within the first two weeks of infection and usually persist for a lifetime in low titres. The presence of these antibodies with the avidity of IgG can be an essential tool to determine the timing of infection (Montoya, 2002; Murata et al., 2016; Villard et al., 2016).

Molecular approaches can be used as a complement of serological tests when the diagnose is unclear by serology and can be used to detect *T. gondii* DNA in various samples (Okay et al., 2009; Robert-Gangneux and Dardé, 2012; Murata et al., 2017; Murata et al., 2020). The most used targets for *T. gondii* are the *B1* multi-copy gene and the 529 bp repeat element with 35 and 200-300 copies in the *T. gondii* genome, respectively (Homan et al., 2000; Ivović et al., 2012; Camilo et al., 2017; Nakashima et al., 2020; Murata et al., 2020). Although molecular tests for the diagnosis of toxoplasmosis are becoming more frequent, they are still controversial, and there is no agreement about the best method or target to be used (Garweg et al., 2011; Maenz et al., 2014; Greigert et al., 2019; Murata et al., 2020). Also, the concentration of *T. gondii* DNA during chronic infection is usually undetectable even when molecular sensitivity tests are performed (Ivović et al., 2012). Consequently, the diagnosis of *T. gondii* infection is still difficult and the development of novel methods with higher specificity and sensitivity is paramount.

Several studies have related the use of microRNAs as new biomarkers in several diseases such as cancer (Wang et al., 2020; Kooshkaki et al., 2020; Di et al., 2020), cardiovascular (Sun et al., 2020), and bacterial diseases (Jia et al., 2014), as well as a non-invasive diagnosis and monitoring method of parasitic disease progression (Hu et al., 2013). A study by Jia et al., 2014 used 60 BALB/c female mice intraperitoneally infected with 10^6^ tachyzoites of RH or ME49 strain per mice to assess the feasibility of using microRNAs as biomarkers of early *T. gondii* infection. Seventy-two hours after infection, the presence of 414 murine microRNAs was evaluated on plasma RNA samples by real-time PCR. The results showed that microRNAs miR-712-3p, miR-511-5p, and miR-217-5p are significantly expressed in mice infected with either *T. gondii* strains (Jia et al., 2014). These authors also found that the up-regulation of these three microRNAs were *T. gondii* specific when compared to similar infections with *P. berghei*, *P. yoelii*, *P. chabaudi*, *C. parvum*, Mouse Hepatitis Virus, and *Staphylococcus aureus*. This observation has drawn attention to the use of microRNAs as early biomarkers of infection in parasitic diseases, which results in their usefulness in the laboratory diagnosis of acute infection, especially before the appearance of IgM antibodies.

The use of a test that could detect *T. gondii* infection in earlier stages would be ideal, mainly during pregnancy, as early treatment of infected mothers seems to decrease the risk of transmission and severity to the foetus improving clinical outcomes (Wallon et al., 2013; Begeman et al., 2017). Expression of microRNAs may precede IgM class antibodies in parasitic diseases since that IgM antibodies may eventually be undetected within the first weeks following infection (Murata et al., 2020). Furthermore, the microRNAs may be biomarkers of high sensitivity and specificity for diagnosis at different stages of infection.

Jia et al., 2014 have shown that three microRNAs were specific for *T. gondii* infection in mice, but there is no study to compare the expression of these biomarkers in humans. The main advantage of using microRNA to diagnose human toxoplasmosis is that they can be found in samples commonly acquired for the diagnosis of *T. gondii* infection such as peripheral blood, amniotic fluid, and aqueous humour, as well as in other types of specimens that can be collected using non-invasive methods like urine, saliva, and others. Also, real-time PCR for detecting specific microRNAs to diagnose human toxoplasmosis is likely the best approach since it usually has higher sensibility and specificity than conventional PCR (Ivović et al., 2012).

To our knowledge, few studies in the literature relate microRNAs to the diagnosis of toxoplasmosis in humans. Our research group investigates the use of four microRNAs (miR-712-3p, miR-511-5p, miR-217-5p, and miR-9-2) for the diagnosis of human ocular toxoplasmosis using blood samples. Our study may contribute to a better understanding of microRNAs’ role during *T. gondii* infections in humans (**Table 1**).

## Final Considerations

The data presented in this revision concerning microRNAs attract the attention for the potential use of these small nucleic acid molecules to explore at least four different aspects of human toxoplasmosis. One of them refers to laboratory diagnosis. Diagnosis methods based on early detection of microRNAs could be an essential tool, especially for detecting microRNA in peripheral blood before IgM antibodies appear. In this context, microRNAs could be explored as biological markers of infection, especially in the acute phase of the disease, allowing the early treatment of human acute toxoplasmosis. This strategy could reduce tissue inflammation and, consequently, the tissue damage in target organs such as the eye and brain.

Another potential application of the detection of microRNA in human toxoplasmosis refers to the tissue targeted by *T. gondii*. Investigations aiming to check if one or more specific microRNA might be expressed in some particular infected tissues would establish a correlation with the clinical form of human toxoplasmosis. Despite the parasite infecting any nucleated cell, it remains in a latent phase, and the majority of individuals remains asymptomatic. However, the human toxoplasmosis clinical manifestation occurs in some target organs and would be appropriate to understand the potential relation between the *T. gondii* and the infected tissues. This strategy could favour the comprehension of new aspects of the host-parasite interaction in human toxoplasmosis.

The observations that some types of microRNA are up-regulated in ocular toxoplasmosis and cerebral toxoplasmosis in HIV patients open opportunity to investigate if this up- or down-regulation correlates specific strains. This strategy could be adding new information on how different *T. gondii* strains modulate the host cells affecting cell mobility through blood, neurological, and ocular barriers. Besides, it would be possible to verify microRNAs also correlates the different ways of *T. gondii* acquisition of the infection by placenta or acquired after birth.

Finally, exploring mi-RNA in the different clinical forms of human toxoplasmosis would clarify how host microRNAs modulate this parasite infection and how *T. gondii* interacts with human hosts.

## Author Contributions

GMFJr, CA, FM, and HL wrote the manuscript. LA and BC drew the table and figure in the manuscript. CB and LM revised the manuscript. All authors contributed to the article and approved the submitted version.

## Funding

This study was supported by Fundação de Amparo à Pesquisa do Estado de São Paulo (FAPESP grants: 2018/09448-8 to GMFJr) and NIH NIAID award number U19AI110819 (to HL). This study was financed in part by the Coordenação de Aperfeiçoamento de Pessoal de Nível Superior – Brazil (CAPES) CMA and LCPA Finance Code 001 and by CNPq 303281/2020-0 (to CCB).

## Disclaimer

The opinions, assumptions, and conclusions or recommendations expressed in this material are strictly those of the authors and do not necessarily reflect the views of FAPESP.

## Conflict of Interest

The authors declare that the research was conducted in the absence of any commercial or financial relationships that could be construed as a potential conflict of interest.
